# Mobility as climate change adaptation in South Africa: Exploring the legal and policy significance of artificial intelligence

**DOI:** 10.4102/jamba.v18i1.2059

**Published:** 2026-05-19

**Authors:** Temitope A. Obisanya, Ademola O. Jegede

**Affiliations:** 1 School of Law, Faculty of Management, Commerce and Law, University of Venda, Thohoyandou, South Africa; 2 Ismail Mahomed Centre for Human and Peoples’ Rights, Faculty of Management, Commerce and Law, University of Venda, Thohoyandou, South Africa

**Keywords:** artificial intelligence, climate adaptation, human mobility, law, maladaptation, planned relocation

## Abstract

This study explores the intersection of mobility, climate change adaptation and artificial intelligence (AI) in South Africa. As climate change impacts mobility patterns, South Africa needs effective adaptation strategies, especially to manage the mobility of internally displaced persons (IDPs) and prevent maladaptation. Artificial intelligence is used to improve lives and livelihoods in such situations, but it raises legal and policy questions. The objective of the article is to uncover the legal and policy implications of AI’s role as a climate technology for adaptation amongst IDPs in South Africa. The article utilises a qualitative desk-based approach to analyse legal frameworks, literature, official records such as parliamentary transcripts, non-governmental organisations (NGO) reports and online repositories on IDPs’ mobility patterns and adaptation, as well as the South African government’s technological interventions to support better adaptation. The findings reveal that AI can improve the planning of IDPs’ relocation. Its tools can improve needs assessment, resource allocation, protection services or infrastructure planning. However, South Africa’s legal frameworks lack specific provisions on AI. Hence, regulations are needed to address AI’s evolving role in climate adaptation in South Africa.

**Contribution:**

The study concluded that with AI policies and laws, maladaptation associated with mobility can be mitigated in South Africa.

## Introduction

According to the Intergovernmental Panel on Climate Change (IPCC) Sixth Assessment Report:

[*C*]limate change is a growing driver of human mobility, with increasing frequency and intensity of extreme weather events like floods, droughts, and storms leading to significant displacement of people globally, particularly in regions with high vulnerability and low adaptive capacity. (IPCC 2023)

Mobility is often used as a coping or adaptation strategy to escape extreme climate impact; however, some mobility typifies and results in maladaptation (Zickgraf 2021; Obisanya and Jegede, 2025). As such, climate mobility and adaptation of displaced persons, in this context, internally displaced persons (IDPs), require urgent, nuanced and systematic research. We argue that artificial intelligence (AI) can aid mobility as an adaptation to extreme climate conditions, thereby reducing maladaptation and creating effective relocation programmes. In this article, ‘climate mobility’ refers to different types of human movements in the context of climate change – migration, forced displacement and planned relocation (Martin 2017). Associating mobility with climate change is contested, as some writings prefer its linkages with disasters, such as earthquakes and tsunamis. Against this preference, Ian Fry, the former United Nations Special Rapporteur on the promotion and protection of human rights in the context of addressing this misconception, submits in his report to the United Nations Human Rights Council that ‘people displaced by climate change should be considered legally and procedurally different to those affected by geological disasters’ (Fry 2023). Likewise, scholars such as Kälin (2023) and Sussman (2024), both international lawyers and experts on climate change, have argued for a distinct legal status and framework for climate-displaced persons. Climate change impacts people globally, unlike natural disasters, which might be limited to a specific geographical area; hence, construing displacement in the context of climate change as a distinct phenomenon is essential, as it has implications for historical causality and international responsibility regarding climate-displaced persons (Fry 2023).

According to the 2022 IPCC report – Impacts, Adaptation and Vulnerability – climate change impacts may broadly be divided into two categories: the sudden or rapid onset of events (floods, storms, heatwaves and wildfires) and more gradual changes or slow onset events (coastal erosion or drought, sea level rise, desertification or ocean acidification). The Report further distinguishes the classification by demonstrating that, for instance, slow-onset events, such as ecological droughts, increase the risk of wildfire, which can lead to human displacement, loss of social ties, a sense of peace and cultural identity, as well as cause migration. Ian Fry has stated that climate mobility encompasses a range of mobility types, such as forced displacement or involuntary immobility, as well as potentially positive types, including autonomous, high agency migration and planned community-led relocation, which can be a good form of adaptation strategy (Fry 2023).

In South Africa, climate-related disruptions to human livelihoods have exacerbated the mobility of IDPs, disproportionately affecting vulnerable populations (Matikinca, Nyamakura & Shackleton 2024). Existing legal and policy frameworks often fail to account for the potential of AI to enhance IDPs’ mobility. As such, we focus on the mobility and adaptation of climate-displaced persons resulting from sudden-onset events caused by climate change, with reference to the use of AI for planned mobility and the relocation of climate IDPs into new environments. Evidence suggests that climate adaptation strategies, such as relocation, may subsequently lead to maladaptation in Africa, thereby making people even more vulnerable (Schipper 2020).

Measures, for example, aimed at assisting the IDPs to cope with displacement associated with climate sudden and slow onsets in South Africa sometimes expose them to rape, extortion, internal conflict for resources and overcrowding (Schipper 2020).

Maladaptation may include infrastructural maladaptation (Adeola 2020), institutional maladaptation (Internal Displacement Monitoring Centre [IDMC] 2024) and behavioural maladaptation (Zaal 2024, para. 10). However, according to the IPCC Report:

[*P*]oor planning is consistently identified as a primary cause of maladaptation to climate change, depicting how deficient relocation/adaptation strategies can worsen vulnerabilities or create new risks, especially for marginalised communities. (IPCC 2022)

This means that such planning should be undertaken from the outset, when the need for relocation is established, through to the eventual integration into the new community or reintegration into the modified previous habitat. In this context, our aim is to uncover the legal implications of AI’s role as a climate technology for adaptation amongst IDPs in South Africa. Consequently, the study attempts to answer the following questions:

- *What are the adaptation gaps and potential in response to climate mobility?*
- *Can the deployment of AI address maladaptation to climate mobility?*
- *Can the existing legal framework aid the regulation of AI as a climate adaptation tool?*
- *How may regulatory measures from other legal systems inform or improve AI as a climate adaptation strategy in South Africa?*

The article uses a qualitative desk-based approach, relying on official records such as parliamentary transcripts, Non-Governmental Organisation (NGO) reports and online repositories on IDPs’ mobility patterns and adaptation, as well as the South African government’s technological interventions to support better adaptation. As such, providing practical and legal solutions to the plight of IDPs in South Africa.

### Impact of climate change on mobility in South Africa

According to the IDMC, between 2008 and 2024, 232 000 people were displaced in South Africa due to 70 sudden-onset events, including flooding, wildfires and storms (IDMC 2024). Historically, those affected persons were initially placed in state-owned shelters, such as community halls and schools (TimesLIVE 2024, para. 3), whilst thousands also found shelter through NGOs, relatives and friends. Some have returned home, depending on the severity of their circumstances (Government Communication and Information System 2024; Johnston et al. 2024).

South Africa’s adaptation policy response to the climate crisis is its ‘National Climate Change Adaptation Strategy (NCCAS)’ – a comprehensive plan that outlines priority areas for addressing climate change impacts, through initiatives like early warning systems (EWS) and community awareness campaigns (The Presidency 2022). The IPCC Report, for example, indicates that the 2015–2017 Cape Town drought underscores the urgency of integrating state and non-state responses to climate change into municipalities’ adaptation and disaster planning to prevent responses with unintended consequences (IPCC 2022). According to IDMC (2024) and the 2024 report on climate change impacts in South Africa (Johnston et al. 2024), people affected by sudden-onset climate change between April 2022 and August 2024 were relocated due to the destruction of their homes and essential facilities. These affected people were either sheltered in churches, schools, and community halls or shared homes with relatives and friends living in areas not affected by the disaster (Department of Health 2022; SAnews 2022, para. 4). This approach predisposed them to human rights abuses, such as exploitation, rape, gender-based violence, limited access to education and loss of cultural identities (Mazibuko 2022, para. 6).

### Adaptation gaps in response to climate change impacts

The current adaptation initiatives have exposed gaps contextualised within the purview of disaster risk management and technological tools for climate adaptation.

#### Gaps in climate adaptation and disaster risk management

The South African government has, over the years, taken steps to address the plight of climate IDPs; these have been supported by NGOs, yet there are gaps in these adaptation strategies and disaster risk management that require urgent attention. In line with these concerns, the United Nations Environment Programme (UNEP) identified gaps in the form of inadequate investment and planning on climate adaptation (UNEP 2023). In South Africa, in light of the climate crisis and the sudden onset of events in KwaZulu-Natal (KZN) (SAnews 2022, para. 2), President Cyril Ramaphosa declared a national state of disaster and made commitments, in collaboration with provincial and municipal structures, to address the crisis (The Presidency 2022). The national government planned to address the climate crisis in three phases: Firstly, offering immediate humanitarian assistance to climate IDPs by catering for their basic needs, secondly, stabilisation and recovery of the IDPs, and thirdly, rebuilding significant infrastructures ranging from critical water and sanitation facilities to vital road networks and bridges (Parliamentary Monitoring Group 2022).

An overview of the three-phase government intervention revealed that IDPs were sheltered in community halls and provided with mattresses, food and hygiene packs (SAnews 2022, para. 6). These goodwill acts, although commendable, generated some concerns as these IDPs complained about issues like the sexual exploitation of their children, exposure to alcohol and drug abuse, lack of privacy in the bathrooms and unique spaces, exposure to extreme cold during the winter period, lack of food and basic needs, inhumane living conditions and constant conflict due to limited resources. It was also alleged that the children had no access to education and basic healthcare (Mazibuko 2022, para. 8); hence, the presumed intervention led to maladaptation. In response to their complaints, in August 2022, the State’s Ad Hoc Joint Committee on Flood Disaster Relief and Recovery paid a courtesy visit to some shelters in KZN and found that the conditions were indeed appalling and that the climate IDPs lived in inhumane conditions (SAnews 2022, para. 7). After investigating these allegations, the government blamed some individuals amongst the climate IDPs for being greedy and taking more than their daily ration and promised to address their plight. The government pledged to intensify its efforts to ensure that climate IDPs had protection from social vices and, especially, that children with disabilities had access to education, healthcare and their basic needs (Ledwaba 2022). By September 2022, however, hundreds of children in shelters were still not attending school, when UNICEF South Africa intervened by providing relief by making recreational toys and learning materials available to the children (Matangira 2022). In response to the allegation, the Department of Human Settlements restated its commitment to move IDPs out of shelters into accommodation units by December 2022 (Macupe 2022). The KZN premier, Sihle Zikalala, made a commitment to build 4396 temporary accommodation units by the end of April 2022 (SAnews 2022, para. 9); however, by September 2025, fewer than 2000 accommodation units had been built, whilst thousands of families were still in temporary or emergency housing, 3 years after the 2022 floods (AllAfrica 2025).

In light of the plight of climate IDPs in South Africa, we argue that the climate change adaptation strategy and the *Disaster Risk Management Act* present gaps in law and practice, particularly in providing shelters, healthcare and education. As indicated by the IDPM reports, climate IDPs in KZN are still housed in churches, schools and community halls. This is a clear indication that the South African government lacks the technical expertise and experience to manage the influx of IDPs (IDMC 2024).

#### Gaps in climate adaptation and risk reduction technology

The IPCC special report on global warming (Hoegh-Guldberg et al. 2018), as well as the IPCC Climate Change report (IPCC 2022) and the IPCC report on Mitigation of Climate Change (IPCC 2014; WGII SPM, p. 20), recognise the role of EWS in supporting climate mobility and adaptation. To address the impacts of climate change, the UNFCCC recommends using climate technology, including EWS (UNFCCC 2024). With the use of EWS as a climate mobility and adaptation tool, the South African Weather Service (SAWS) currently utilises an Impact-Based Severe Weather Warning System (ImpB-SWWS) intended to warn the public of possible adverse climate impacts, a shift from using the traditional Severe Weather Warning System (SWWS) (SAWS 2023). An ImpB-SWWS may inform people of impending climate crises and indicate their level of impact; however, some of the differences with the use of the ImpB-SWWS are the operational speed, length of forecasting period, forecasting accuracy, the dissemination process, response capacities, as well as the level of preparedness to address the crisis (SABC 2024, para. 5; SAWS 2020).

Re-affirming the technology gaps for climate EWS and adaptation, the UN Secretary-General, António Guterres, in 2022, during COP 27, called for a global effort to ensure that the four pillars for an effective climate EWS, contained in the EW4All on Earth initiative, are implemented by different organisations (United Nations Disaster Risk Reduction [UNDRR] 2022). These pillars are examined in the ensuing subsections.

### Pillar 1 – Disaster risk knowledge

Whilst tasked with the mandate to implement Pillar 1 of the EW4ALL initiative, the UNDRR uncovered significant gaps in terms of risk information and assessments worldwide, in that ‘less than half of the countries with existing EWS have access to appropriate disaster risk information, and even fewer have national legislation and regulatory frameworks for emergency response’ (UNDRR 2022). An overview of South Africa’s response to the Pillar 1 demonstrates that the country does have an EWS, as well as a couple of disaster management frameworks, such as the *Disaster Management Act of 2002* and the National Climate Change Response (NCCR) policy. The EWS and the regulatory frameworks, however, are fraught with gaps and inept implementation processes. To address these concerns, Pillar 1 aims to boost global risk knowledge and incorporate it into EWS, thereby advancing stakeholder coordination innovation and empowering decision-makers and vulnerable communities to understand, identify and respond to climate risks (UNDRR 2022).

### Pillar 2 – Detection, observations, monitoring, analysis and forecasting of hazards

Pillar 2, coordinated by the World Meteorological Organisation (WMO) and other cohorts, aims to shed light on the evolving nature of climate change so that governments, communities and individuals can be prepared to respond appropriately to climate crises (WMO 2023). To actualise this, it is suggested that the EWS must be human-centric, affording humans ample time and opportunity to prepare and respond to climate crises (WMO 2023). An evaluation of South Africa’s performance on Pillar 2 was brought to light by the recurrence of climate crises in KZN, Western Cape and other provinces. The recurrence of climate crises and the country’s ineffectiveness in managing them indicate that much still needs to be done. As indicated in the former sections, SAWS currently utilises ImpB-SWWS, which is not adequate for early detection, observations, monitoring, analysis and forecast – processes which aid in viable responses to climate crises.

### Pillar 3: Warning dissemination and communication

Pillar 3 focuses on the systematic warning strategy and communication of climate risk to individuals and communities. This initiative was led by the International Telecommunication Union (ITU) (United Nations 2023). The ITU identified gaps in dissemination and communication within EWS, as well as in legal frameworks that could facilitate the process (ITU 2025). In response to these gaps, it was suggested that dissemination of information should not be a ‘one-size-fits-all approach’ (ITU 2025). Hence, member nations, depending on their financial capability, should address their particular communication challenges. This can be achieved by strengthening existing community-based infrastructures and local feedback mechanisms, such as radio, television, social media, sirens, mobile phones and satellite.

In South Africa, SAWS mainly uses radio and television to disseminate warnings of impending climate hazards. (SAWS 1994). Television and radio are valuable means of dissemination; however, SAWS are yet to thoroughly utilise other forms of mobile communications as recommended by the ITU (ed. Department of Communications and Digital Technologies 2019).

### Pillar 4: Preparedness to respond

Led by the International Federation of Red Cross and Red Crescent Societies (IFRC), Pillar 4 evaluates how governments, non-governmental agencies, communities and individuals undertake anticipatory responses to climate crises (IFRC n.d.). An anticipatory response is an action taken before a predicted crisis occurs to prevent or reduce its potential impact (IFRC n.d.). Examples of these actions include – early evacuation, reinforcing homes, distributing health protection kits, setting up mobile cooling centres and distributing cash (IFRC 2022). According to the IFRC, most governments and NGOs provide humanitarian support after the climate crisis, rather than utilising AI EWS that can forewarn, support proper planning and help manage the impending crisis (IFRC 2024).

In summary, juxtaposing South Africa’s existing adaptation and risk-reduction technology strategies with the findings and recommendations of the four Pillars of the EW4LL initiative indicates that South Africa does have in operation a combination of Pillars 1, 2 and 3. This assertion is based on the country’s warning systems, which can monitor and predict climate hazards, communicate their impacts and disseminate them to communities and the country timeously. These warning systems, although available, are not viable for early planning, evacuation or relocation of IDPs. For example, other EWS, such as the FuXi-Subseasonal model, has the capacity to significantly increase the prediction period for extreme weather from 30 days to 36 days; however, predicting potential climate disaster events as early as possible, and gaining more time for response and mitigation measures (Chen et al. 2024; Yusha 2024, para. 8), are absolutely imperative. According to the SAWS Report (46), releasing 7-day forecasts might not be sufficient for South Africans to plan and respond to the impending climate crisis. As such, according to the SAWS Report for 2023/2024, early warnings of snowfall occurred – from 08 July 2023 to 10 July 2023 over the Western Cape; KZN on 09 July; Mpumalanga and Southern and Central parts of Gauteng on 10 July 2023 – were only issued by SAWS on 08 July 2023, through the media. Similarly, early warning of the stormy weather that occurred over Central, Southern and Eastern parts of South Africa on the 23 December to 26 December was only issued on 22 December 2023 (SAWS 2023). This indicates that relying on the existing early warning system would make it a mammoth task to plan the mobility and relocation of IDPs, compared to other climate technologies that can issue early warnings 30 days to 36 days before the event. It is worth noting that communities may resist evacuation or relocation; therefore, concrete plans and policies must be implemented to encourage relocation or justify the government’s actions or inactions if the community or individuals suffer harm due to a climate hazard. Petteri Taalas, the Secretary-General WMO, in the Executive Action plan for 2023–2027, indicated that:

Early warnings save lives and provide vast economic benefits, with just 24 hours’ notice of an impending hazardous event, having the potential to cut the ensuing damage by 30 per cent. (UNDRR 2022)

The Global Commission on Adaptation found that ‘spending just $800 million on such systems in developing countries would avoid losses of $3–16 billion per annum’ (United Nations 2023).

South Africa’s approach to addressing the climate crisis lacks Pillar 4, representing a significant gap in the climate adaptation and risk management strategies. As alluded to by the IFCR, although the government and NGOs extend humanitarian assistance to support individuals and communities affected by disasters, the support would be more effective if well-planned before a climate hazard. The pains and neglect individuals and communities have to go through whilst in transit and trying to adapt to their new environment, according to the IFCR, can be mitigated by having the proper early warning climate technology to address climate change and associated crises (IFRC 2022; Ncube et al. 2016).

### Artificial intelligence early warning system as a potential climate change adaptation tool

As indicated, the IPCC AR5, IPCC {SR15} and IPCC Special Report on Climate Change and Land (SRCCL) (Olsson et al. 2019) highlight the significance of AI EWS as a key climate adaptation technology that can aid in reducing climate-related disasters. Artificial intelligence has been projected to be capable of performing tasks, such as problem-solving and planning for effective climate adaptation (Shabbir & Anwer 2018). Aside from early warning and climate impact estimates, governments and NGOs such as the Red Cross and Gift of the Givers are considering AI’s potential to predict and simulate responses to various climate emergencies to aid climate adaptation, in line with Pillar 4 of the EW4LL initiative (IFRC 2023). This suggests that AI can play a significant role in climate adaptation, such as supporting Earth Observation to identify accessible roads during disasters, such as floods; humanitarian efforts to identify the most vulnerable; conducting needs assessments; and resource allocation (one of the main concerns in climate adaptation).

Also, as an adaptation tool, AI can improve shelter planning for short- and long-term adaptation strategies. AI tools, such as AI-driven smart-home systems (AIDSHs), can reduce energy usage by adjusting home appliances and monitoring and controlling lighting, temperature, security and air conditioners to adapt to extreme weather (Dąbrowska 2024). The AI system can suggest for example – energy-efficient appliances so that users may adjust and/or adapt to any potential climate hazards comfortably (Cyanergy 2024); predicts maintenance schedules for appliances for optimum usage (Dalzochio et al. 2020); optimises water distribution from the water grill, through detecting and minimising leaks and waste (Sakti et al. 2023); suggest smart electricity grids, which are capable of conserving energy and aiding adaptation to climate risk (Khaleel et al. 2024).

In this context, AI systems can aid in IDPs’ effective adaptation and resilience in a new environment through AI EWS and climate AIDSHs. As noted earlier, there are growing concerns about the government’s ability to build accommodation for IDPs. This presents a good opportunity for the government to develop an innovative AI home community, where IDPs affected by climate change will be housed rather than in churches, township halls, or squatter camps. The AI intelligent-home systems, integrated with other technological systems such as biogas, can effectively manage human waste, which is then converted to energy for cooking and warmth during winter. Artificial intelligence inculcated in building plans can also help regulate temperature, monitor unsolicited activities and pre-empt heinous crimes such as rape and drug peddling, which are issues of great concern to the IDPs. Artificial intelligence systems can also help continue the education of children of climate change IDPs at the allocated centres through video and interactive learning.

### Good practices of artificial intelligence as a response to climate adaptation management gaps

#### Artificial intelligence early warning system

Recently, scientists have unveiled climate AI technologies, such as sub-seasonal forecast tools (UNCC 2024) and FengWu-GHR (Rasp et al. 2024), that can predict potential climate sudden-onset events as early as possible, thereby providing more time for mitigation and adaptation (Langrand 2024). Comparing these AI tools with the existing SAWS, EWS shows a significant gap in prediction and accuracy in the detection of climate sudden-onset events.

#### Artificial intelligence climate adaptation management tools

AI-driven smart-home systems can be used to plan IDP shelters and accommodations to aid in better climate adaptation. The AIDSH system may be used as a smart electricity and water grid to manage heat, lighting and water appliances, conserving energy and thereby facilitating resilient adaptation (Khaleel et al. 2024). Examples of such AIDSH systems are LG’s Smart ThinQ, Daikin’s Altherma, Carrier’s Cor, Ariston’ Net, Rheem’s EcoNet and Stiebel Eltron’s SENZ. Other AI climate adaptation tools, such as EUMigraTool (Langrand 2024), can be used in the context of IDPs’ mobility and relocation, hence, also help manage adaptation and resettlement of IDPs in South Africa. These AI tools prevent maladaptation to the new environment by, for example, preventing overcrowding; aiding facial recognition; controlling food and water distribution in resettlement camps; and capturing illegal activities, such as theft, rape, drug peddling, gender-based violence, and other vices, thereby aiding easy prosecution. This article, therefore, suggests that AI has the potential to address IDPs’ challenges in South Africa (World Economic Forum 2022).

This article is not oblivious to the legal implications of deploying AI as an adaptation tool, such as bias, invasion of privacy, security issues and the potential to contribute to greenhouse gas (GHG) emissions (UNFCCC 2024). The application of AI as an adaptation tool to aid IDPs must be established within the framework of international law. As such, AI-driven tools such as facial recognition or predictive policing therefore require strict legal safeguards to ensure proportionality – that state measures are necessary and not excessive relative to legitimate aims like public safety. Courts have reinforced this principle in cases such as Digital Rights Ireland v. Ireland (Court of Justice of the European Union 2014), which highlight the dangers of mass surveillance without adequate oversight. As it stands, the potential future impact of AI as an adaptation tool is unclear; nevertheless, if AI is developed and deployed responsibly, for example, by developing energy-saving AI tools, just like energy-saving bulbs, the future can be promising. Hence, this article argues for a regulatory framework whilst deploying AI as an adaptation tool in South Africa, to safeguard the future.

#### Artificial intelligence potential in key regulatory frameworks in South Africa

As AI technologies continue to evolve and permeate human lives and livelihoods, arguably, AI can be used to aid climate adaptation; however, its development and deployment must be done responsibly. None of the existing international or domestic instruments has specifically referenced AI as a climate adaptation technology. However, there are evident provisions that indirectly support the use of AI to aid climate adaptation.

#### The United Nations High Commission for Refugees guiding principles on internally displaced persons

The UNGP is an international instrument that identifies the rights and protection of IDPs. It also aids the IDPs in all mobility, relocation and social reintegration phases; although the UNGPs are not binding instruments, they reflect and are consistent with international human rights, such as civil and socioeconomic rights. It may also be argued that the UNGP currently constitutes customary international law (Lwabukuna 2012). Principle 3, read with 7(2), 8, 18 and 23 of the UNGP, places a burden on governments to protect and provide humanitarian services and proper accommodation for IDPs, and to ensure that such displacement does not violate the civil and socioeconomic rights of those affected. Also, the UNFCCC (1992), UN Paris Agreement 2015 (UN 2015) and the UNDP on climate change adaptation (UNDP 2024) emphasise the need for climate justice and shared responsibility in addressing climate change. Arguably, countries that are historically responsible for climate change and its consequences should lead in providing necessary services. Even if it is argued that the UNGP principles are not binding, they are consistent with international human rights obligations, which require states to respect, protect, promote and progressively fulfil socioeconomic rights.

The UNGP makes no specific reference to AI as a potential technology to achieve its objectives; nevertheless, in principle, it can be argued that the UNGP supports the use of AI. This is because, for example, one of the primary uses of AI in the context of sudden-onset climate events is to facilitate climate adaptation and enhance protection for IDPs. In the context of the UNGP, AI can be deployed in ways that aid the actualisation of IDP rights.

For example, Principle 7 connotes that:

- Before any decision requiring the displacement of persons, the authorities concerned shall ensure that all feasible alternatives are explored to avoid displacement altogether. Where no other options exist, all measures shall be taken to minimise displacement and its adverse effects.
- The authorities undertake that such a displacement shall ensure, to the greatest practicable extent, that proper accommodation is provided for the displaced persons, that such displacements are effected in satisfactory conditions of safety, nutrition, health and hygiene, and that members of the same family are not separated.

In context, Principle 7 holds that state institutions and authorities explore feasible alternatives to avoid displacement, which might never have occurred if the right technology had been deployed. As such, the UNGP suggests, in its totality, the use of the latest technologies, such as AI EWS and AI tools, to aid climate mobility and the resettlement of IDPs. As a result, it is suggested that AI–AI-informed climate mobility initiatives and laws in South Africa should prioritise the protection of IDPs’ right to life, health and medical care; adequate food, shelter, security, dignity, violence, as well as protection from any form of discrimination and exploitation using AI technologies.

### The African Union Convention on the Protection and Assistance of Internally Displaced Persons in Africa (Kampala Convention) of 2009

The Kampala Convention (African Union 2009) is yet another international instrument that aims to cater to IDPs’ needs. Article V of the Kampala Convention, like the UNGP, obliges States Parties to protect and assist IDPs. South Africa is not a signatory to the Kampala Convention; however, the government is bound to protect its citizens and fulfil their fundamental human rights, regardless of their status as displaced persons.

Article IV refers explicitly to EWS technology to mitigate the impact of climate change. Article IV provides that:

The States Parties shall devise early warning systems, in the context of the continental early warning system, in areas of potential displacement, establish and implement disaster risk reduction strategies, emergency and disaster preparedness and management measures and, where necessary, provide immediate protection and assistance to internally displaced persons.

In other words, scientific evidence suggests that AI technology can be used as EWS and aid preparedness and management of the climate crisis (UNFCCC 2024). It is paramount, therefore, that state governments and Institutions use AI technology to actualise the provisions of the Kampala Convention.

### International Covenant on Economic, Social and Cultural Rights

Socioeconomic rights are the second generation of rights, which originated from the International Covenant on Economic, Social and Cultural Rights (ICESCR). Articles 11, 12 and 13 of the ICESCR recognise everyone’s fundamental rights, such as the rights to housing, health, and education, respectively, and obligate the state to take appropriate steps to achieve these rights. These rights are adopted and enshrined in the South African Constitution (SA Constitution 1996). The purpose of engraving these rights in the SA constitution is to mandate the government to formulate policies towards the actualisation of socioeconomic rights, which will equip courts to intervene when such rights are infringed upon or implemented unsatisfactorily. In theory, these rights allow citizens of signatory nations, climate change IDPs included, to *inter alia* demand basic socioeconomic rights from the government.

The ICESCR does not specifically refer to AI technology in its provision, although, it can be deduced that the provisions are intended to imply also the use of AI technology to actualise the rights of IDPs. Article 15(1), for example, provides that state parties to the present Covenant are to recognise the right of everyone to enjoy the benefits of scientific progress and its applications. The ICESCR encourages the use of science, which arguably includes AI technologies, to achieve the full realisation of socioeconomic rights. The Committee on Economic, Social and Cultural Rights (CESCR) General Comment No. 25, (United Nations 2020) and the International Justice Resource Center (IJRC 2020), respectively ‘clarifies that individuals have the right to not only benefit from scientific progress but also to actively participate in it’.

### The South African constitution

Moving to domestic instruments, the SA constitution is one of the most profound and progressive constitutions in the world, being that it is founded on dignity, equality and freedom. These rights are also extended to IDPs as they are citizens of South Africa. The government, under the constitution, is obliged to respect and protect the dignity of IDPs by providing them with the right to proper housing, education and a clean and healthy environment. Sections 24, 26, 27 and 29 of the SA *Constitution*, respectively, provide as follows:

- ‘Right to an environment that is not harmful to their well-being and to have it protected for the benefit of present and future generations’.
- ‘Right to have access to adequate housing, which the government must take reasonable legislative and other measures within its available resources to achieve the progressive realisation of the right’.
- ‘Right to health care, water and social security’.
- ‘Right to education’.

The inclusion of the rights to health, education and housing in the constitution, however, often does not translate into action, especially in the context of IDPs; hence, it is argued that the government should provide the IDPs with basic rights, by developing AI systems that support climate change adaptation efforts, that will aid the actualisation of the above-mentioned rights. The South African government is a signatory to the socio-economic rights and is bound to fulfil measures that will enable IDPs to enjoy these rights, including using technology such as AI to promote environmental education, security and access to basic health care and information. The CESCR’s General Comment No. 25 (United Nations 2020) also prescribes that States parties should ensure that everyone has equal access to science applications, particularly when they are instrumental for the enjoyment of other economic, social and cultural rights. In essence, states are to ensure that AI technologies and tools to address climate crisis are human-centric and prioritise fundamental rights.

#### South African Disaster Management Act

The primary aim of the DMA (Republic of South Africa 2002) is to establish a comprehensive, integrated and coordinated disaster management framework to proactively address climate risk, mitigate climate disasters and support post-disaster recovery by establishing national, provincial and municipal disaster management centres. The DMA does not explicitly mention climate change IDPs in its provision, although, it can be argued in terms of Section 27 (1)(2) of the DMA, read with Sections 24, 26, 27 and 29 of the SA constitution, that the government must provide the basic needs of the IDPs and to take other steps that may be necessary to prevent an escalation of the disaster.

It is worth noting that, as previously discussed, the entire sub-paragraphs of Section 27 of the DMA are relevant, yet the government has not taken the necessary measures to address mobility and disaster management issues affecting the IDPs. Section 27(2)(m) may be read as utilising AI EWS to facilitate responses to the climate crisis and to support disaster risk management for post-disaster recovery and rehabilitation. As such, Section 27(2)(n) should be read as taking steps, such as integrating AI into other technologies like smart-home systems and biogas technology; taking such steps is crucial to prevent maladaptation. Finally, Section 27(2)(o) can be construed as requiring the Minister of Cooperative Governance and Traditional Affairs (CoGTA), in cooperation with other cabinet members or climate change experts, to seek ways to facilitate international assistance/finance for the development and deployment of AI for climate mitigation and adaptation. The CoGTA and other relevant stakeholders, in addition to sourcing funds locally, should take a bold step to engage those historically responsible for the current climate change and its consequences, as well as to facilitate the adoption of AI as a climate mobility and adaptation technology in South Africa. In summary, this article suggests integrating AI into existing laws, particularly the DMA.

#### South Africa’s national climate change adaptation strategy

The NCCAS makes no specific reference to AI in its plan to aid climate adaptation; however, the framework suggests strategic intervention to promote and invest in research on the most effective adaptation responses to different climate change impacts (Republic of South Africa 2019). It further recommends establishing programmes to encourage research into new climate change adaptation technologies and innovations, thereby supporting climate change adaptation in South Africa.

It should, however, be noted that, although the NCCAS was developed to offer policy guidelines for the climate change crisis, the strategy itself was not associated with a specific climate change policy; hence, the crafting of a specific national climate change policy, which will be discussed in the ensuing Section.

#### National climate change response (White Paper)

The NCCR policy provides a foundation for South Africa’s climate change response, prioritising sustainable development, economic growth and human well-being (Republic of South Africa 2011). Like the NCCAS, the NCCR has no AI-specific strategies and does not adequately address the potential of AI to aid climate adaptation.

The NCCR policy also lacks a clear AI regulatory framework for climate change, let alone for those displaced by the climate crisis. It nevertheless encourages the use of technology, such as EWS, to address the climate change crisis (Garland 2014). Hence, the integration of AI into technological tools such as EWS aids climate adaptation. In that light, it is recommended that national, provincial and local governments should integrate AI policies into their climate adaptation strategies. As indicated in the NCCR policy and the NCCAS, adequate funding for climate change adaptation technologies and innovations is paramount for effective planning and coordination of an integrated response to climate change. According to Paragraph 11 of the NCCR policy, the government, in pursuit of a long-term funding framework for climate finance, must – amongst other strategies – promote fair, transparent and timely access to international and domestic resources/fundings for both mitigation and adaptation actions and enable the local development finance institutions to create and implement long-term climate-resilient investment programmes, such as AI EWSs.

In general, Paragraph 11 of the NCCR policy, read with Sections 24, 26, 27 and 29 of the SA constitution and Article 11, 12 and 13 of the ICESCR, requires that states, in this context, the South African government, to progressively make provisions for the actualisation of socioeconomic rights of its citizens, including climate change IDPs. This view is further strengthened by international frameworks such as the Kampala Convention, UNGP and the ICESCR, which address the plight of climate change IDPs. Section 233 of the SA *Constitution* provides that:

When interpreting any legislation, every court must prefer any reasonable interpretation of the legislation that is consistent with international law over any alternative interpretation that is inconsistent with international law.

An overview of Section 233 read with Section 39 of the SA *Constitution* makes it clear and mandatory that courts, tribunals and forums must consider international laws when interpreting the Bill of Rights. South Africa already has policies and judicial precedents that bind the government to provide affordable housing for indigent citizens, which have hitherto been complied with. The rights of IDPs, as set out in international instruments and supported by National frameworks, are to be respected, protected, fulfilled and promoted through evolving technologies, such as AI EWS and tools.

The interpretation of Section 27 (1)(2) of the DMA and the NCCR policy must be consistent with provisions, principles and guidelines as prescribed in international laws, which proffer that AI technologies can be used to mitigate and aid adaptation to climate change in South Africa. It also advocates amending the NCCR policy to promote innovation and the deployment of AI climate technology tools, whilst ensuring safety, fairness and transparency in their development and deployment.

## Conclusion and recommendations

Climate change poses a significant threat in South Africa, including increased displacement of the affected population. Whilst most IDPs use mobility as a form of coping strategy to escape the climate crisis, there is a need to ensure that such mobility does not translate to other forms of maladaptation. There is some evidence that AI could make significant additional contributions to adaptation strategies in the years ahead. Artificial intelligence is not the ultimate panacea for climate change; however, studies have shown that AI EWS, compared to the conventional EWS, can better predict climate-change crises, enabling proactive planning, evacuation and relocation of IDPs. In addition, AI can aid better adaptation regarding humanitarian support and infrastructural design. That said, integrating AI with other climate technologies raises both legal and policy considerations, given AI’s dual role as both a potential climate adaptation technology and a potential environmental threat. As a result, regulatory frameworks must be put in place to guide the development and deployment of AI as a climate mobility and adaptation technology. This article, hence, recommends using AI for climate mobility and adaptation in South Africa.

### The South African government should create an artificial intelligence task team

This group will include experts from private and state institutions, academia and the AI community, who will guide AI deployment and regulations. Currently, the government has a series of forums – parliament, the Inter-Ministerial Committee on Climate Change (IMCCC), civil societies, the Forum of South African Directors-General clusters, the Intergovernmental Committee on Climate Change (IGCCC) and the National Disaster Management Council – which can be deployed for this task. Any of these groups or cohorts can guide the development of AI climate policies to regulate the deployment of AIDSHs and EWS; these policies will address issues of liability and accountability, human rights concerns and deployment as a climate adaptation tool. The team, amongst its duties, should be able to define AI terminologies and concepts and set safety and security standards for deploying and using AI. In collaboration with the government, the team should mobilise the resources necessary for climate mitigation and adaptation. This may be in the form of financial resources, technical cooperation and technology transfers at domestic, sub-regional, regional and international levels.

### Public consultation and engagement

It is paramount that the AI task team engage with civil society, private and public institutions, experts and international institutions to gather input on AI regulations from diverse perspectives.

We recommend that SAWs upgrade existing warning systems and collaborate with other stakeholders to increase response time for mobility planning and adaptation. Although SAWs have notably disseminated early warning information via radio and television stations, it would be more worthwhile to also use SMSs, as recommended by the ITU.

Finally, it is further recommended that Section 27 of the DMA and Paragraph 11 of the NCCR policy be read to imply that the South African government shall use or consider the use of AI EWS and AIDSHs to aid mobility and adaptation/resettlement of IDPs in their new environment or housing, rather than in community halls, schools, churches, shacks, squatter camps and other dehumanising environments. As a matter of policy, it would be prudent to set aside resources for long-term planning for climate mobility and the adaptation/resettlement of IDPs, as contemplated in the NCCR policy. As promised, the South African government should fulfil its commitment to progressively build the 4,396 temporary accommodations promised to KZN’s IDPs in Ilembe District, Ugu and eThekwini. Such accommodation should reflect the smart-home systems, integrated with other technological systems, such as biogas.
